# Intestinal microbiota modulation at the strain level by the olive oil polyphenols in the diet

**DOI:** 10.3389/fnut.2023.1272139

**Published:** 2023-10-04

**Authors:** Natalia Andújar-Tenorio, Antonio Cobo, Ana Mª Martínez-Rodríguez, Marina Hidalgo, Isabel Prieto, Antonio Gálvez, Magdalena Martínez-Cañamero

**Affiliations:** ^1^Área de Microbiología, Departamento Ciencias de la Salud, Universidad de Jaén, Jaén, Spain; ^2^Departamento de Microbiología, Universidad de Granada, Granada, Spain; ^3^Departamento de Estadística e Investigación Operativa, Universidad de Jaén, Jaén, Spain; ^4^Área de Fisiología, Departamento Ciencias de la Salud, Universidad de Jaén, Jaén, Spain

**Keywords:** intestinal microbiota, enterococci, virgin olive oil, polyphenols, high fat diets

## Abstract

**Introduction:**

Previously we have reported a r16S gene next generation sequencing study on the effect of high fat diets in the intestinal microbiota using a murine model. However, many important microbial traits occur at strain level and, in order to detect these population changes, culture-dependent approaches need to be applied. With this goal, we decided to study a very well-known commensal genus, *Enterococcus*, and therefore, intestinal enterococci methodically isolated during the above-mentioned experiment were analyzed.

**Materials and methods:**

A collection of 75 distinct enterococcal strains isolated from feces of mice fed a standard diet or high-fat diets enriched with butter, refined olive oil, or extra virgin olive oil and after 0, 6 or 12 weeks of diet, were genetically and phenotypically characterized in search of virulence factors, biogenic amine production and antibiotic resistance. All strains were tested for the susceptibility *in vitro* to two virgin olive oil polyphenols, oleuropein (the bitter principle of olives) and hydroxytyrosol (derived from oleuropein by enzymatic hydrolysis and responsible for the high stability of olive oil).

**Results:**

No drastic polyphenol effect was found except at high concentrations. However, when carrying out a comparative statistical study in the 75 strains of the collection according to the different diets, we have detected significant differences between the strains isolated from mice fed with a diet enriched with virgin olive oil and the rest of the diets. EVOO strains also presented less resistance to antibiotics and a more beneficial profile overall.

**Discussion:**

These results support the prebiotic role of polyphenols, showing how they are able to modulate the set of strains that comprises a genus in the gut, allowing them to adapt to a changing environment in the host’s intestine and possibly exerting effects on its physiology.

## 1. Introduction

Different types of diet modulate the intestinal microbiota since birth and its composition is sensitive to the proportion of dietary constituents, responding differently to the host’s nutritional profile (1). This influence exerted by the diet is linked to some diseases or pathological situations such as obesity (2–4). In this context, high-fat diets have been proved to result in overweight and obesity, apart from producing negative effects by acting on the gut microbiota such as inflammation (5, 6), higher risk of developing intestinal diseases as Crohn’s disease (5, 6), or promoting intestinal hyperpermeability (7). However, these effects depend on the type of fat and the degree of saturation in the fatty acids. Our previous studies, where we used different high fat diets to compare the influence of these in mice, showed evidence supporting a link between specific diets, some bacterial taxa and different physiological parameters. We reported studies comparing the influence of virgin olive oil (EVOO) and butter (BT), both using genotyping methods (8) and massive sequencing (9). Furthermore, we showed the possible effect of virgin olive oil polyphenols by comparing EVOO and refined olive oil (ROO) diets (10), probably due to the antimicrobial activity of its components both on bacterial taxa *in vitro* (11) and murine models (10).

The technologies used in these studies, such as metagenomics, facilitate the study of the intestinal microbiota by allowing extensive knowledge of its composition without the need to grow the different taxa that compose it. This is essential since the vast majority of them are difficult to cultivate or have never been isolated. However, this does not imply that we renounce to culture-dependent studies, since these are the ones that really allow us to comprehend a bacterial group. In addition, many of its most important characteristics occur at the strain level. This is the case of virulence factors, resistance to antibiotics or the capacity for genetic transfer and intercellular communication, which can mean the evolutionary adaptation that allows a certain strain to outcompete its competitors, establishing itself in a determined environment, and damaging or favoring the corresponding host accordingly. And this information can never be obtained through massive sequencing, which hardly reaches the species level, but rather requires the isolation and characterization of the corresponding strains in the laboratory.

Therefore, the intestinal microorganisms will use nutrients provided in the diet, competing and adapting to the available resources, producing different changes at strain-level. They will have to defend themselves against different toxic chemical products or aggressive nutrients such as antimicrobial peptides, which will impel these bacterial groups to develop different defense mechanisms such as antibiotic resistances, expression of virulence factors or production of biogenic amines, which cause important clinical consequences in the host and in our health. Specifically, biogenic amines are biologically active nitrogenous organic compounds, found in different food and beverages, *via* the enzymatic decarboxylation of their precursor amino acids by certain microorganisms (12). Its presence in the organism can cause adverse toxicological reactions and intoxications harmful to health (13), being tyramine and histamine the most common ones found in foods (14–16). Indeed, The European Food Safety Authority (EFSA) deems them to be the most toxic of all biogenic amines (17).

In this sense, the genus *Enterococcus* is one of the best studied intestinal genera, especially with respect to the presence of different harmful genetic traits. *Enterococcus* is characterized by being commensal intestinal bacteria, being a bacterial group widely distributed in different environments and having a great capacity to survive in adverse environmental conditions and heat treatments (18). They are lactic acid bacteria used as probiotics (19) and in food fermentations (20). However, this bacterial genus is found in the oral-fecal route and they represent important nosocomial pathogens. For this reason, their use and security are controversial (19). Therefore, *Enterococcus* is a good example to study how strains with different harmful traits can be modulated in the intestine depending on how nutrients in the diet affect them. Consequently, we already reported some studies on different intestinal strains of this genus isolated after diverse diets (21).

In the present study we report the effect of two olive oil polyphenols on a collection of 75 genetically unique enterococcal strains isolated from feces of mice fed with just standard chow, or chow enriched with EVOO, ROO and BT as described previously (8). Twenty-five of these strains had never been characterized, so we also report these results here and analyze them together with the results previously reported for the rest of the collection (21) in order to increase the statistical robustness and discuss the possible connotations of the data obtained.

## 2. Materials and methods

### 2.1. Isolation and identification of bacterial strains

The 75 bacterial strains used in this study belong to a collection obtained in a previously published experiment (8) where 12 male SwissWebster ICR (CD-1) mice (Harlan Laboratories) were divided into four groups and fed for 3 months with a standard chow (SD, standard laboratory mice diet A04, 3% fat, Panlab, Barcelona, Spain) or one of three high fat diets (standard chow supplemented with 20% of extra-organic virgin olive oil (EVOO), refined olive oil (ROO), or butter (BT)). All experimental procedures were performed in accordance with the European Communities Council Directive 86/609/EEC and reviewed and approved by the Bioethics Committee of the University of Jaén. Fecal samples were obtained in the first, sixth and twelfth week of the experimental period, and in order to select for enterococci colonies, serial dilutions of feces were used for plating on Tryptic Soy Agar (Scharlab, Barcelona, Spain) plus 100 mg/L polymyxin B, and on Bile Esculin Agar (Scharlab). Putative enterococci were selected according to observation of colony characteristics and cell morphology, Gram staining, catalase and oxidase production, growth in the presence of 6.5% NaCl, at pH 9.6, growth at 10°C and 45°C, and growth and esculin hydrolysis on bile-esculin agar (Scharlab). Genetic identification at species level was done by species-specific PCR and 16S rDNA sequencing as described elsewhere (22). Fifty strains were identified and described previously (21) and the rest, 25, where studied ever since and are described for the first time in this work.

### 2.2. RAPD-PCR amplification

Genotyping using randomly amplified PCR was carried out after the strain DNA was extracted. For this, the primer M13 was used as described in a previous study (20). Each strain gave a distinct pattern of amplified bands and all of them were analyzed in a single dendrogram using FINGERPRINTING II InformatixTM Software.

### 2.3. Growth in the presence of polyphenols

Overnight cultures at 37°C of each of the enterococcal strains were used to inoculate TSB medium with oleuropein (Sigma) or hydroxytyrosol (Sigma) at final concentrations of 15.7 μg/mL, 31.25 μg/mL, 62.5 μg /mL, 125 μg/mL, 250 μg/mL and 500 μg/mL. All inocula as well as controls for each concentration were carried out in triplicate and incubated again at 37°C for 24 h, after which growth was monitored by measuring absorbance at 595 nm. Percentage of growth was obtained with respect to inoculated controls without polyphenols (growth measurements at each polyphenol concentration were divided by growth measurements at 0 mg/mL and multiplied by 100).

### 2.4. PCR amplification of virulence factors

Specific PCR reactions in duplicate were carried out to determine the presence of several genes involved in different virulence factors: enterococcal surface protein (*esp*), the aggregation substance (*agg*), gelatinase (*gelE*), sex pheromones (*cob, cpd* and *ccf*), cell wall adhesion (*efaAfs* and *efaAfm*) and the expression of cytolysin (*cylA, cylB* and *cylM*), as previously described (21).

### 2.5. Biogenic amine production

PCR amplification in duplicate was carried out for the detection of biogenic amine production. Specifically, the presence of the amino-acid decarboxylase genes *hdc* (histidine decarboxylase), *odc* (ornithine decarboxylase) and *tdc* (tyrosine decarboxylase) were analyzed as described before (21).

### 2.6. Antibiotic resistance

In duplicate, overnight strains were inoculated in ATB STREP EU (08) strips (BioMérieux, Marcy-l’Etoile, France) to determinate the susceptibility of enterococci to different antibiotics, following the manufacturer’s instructions.

### 2.7. Statistical studies

In order to assess whether the variable “species” has the same distribution across the various categories of the “diet” variable and the “time” variable, we conducted the exact Fisher’s test for contingency tables. This test was chosen because some of the expected counts were below 5, rendering the chi-square test of homogeneity unsuitable. Similar analyses were performed to investigate the potential influence of “diet” or “time” on the variables “*tdc*” and “*esp*.”

To examine the equality of the distribution of the variable “proportion of antibiotic resistance” across different types of diets, we employed the Kruskal-Wallis test. The same procedure was employed to assess the equality of the distribution of “proportion of antibiotic resistance” across different time periods as well as the growth of the strains in presence of two different polyphenols at several concentration levels. Upon detecting significant differences, we conducted post-hoc Dunn’s test for pairwise multiple comparisons, adjusting the *p*-values using Bonferroni’s correction.

All analyses were conducted within the R environment for statistical computing.

## 3. Results

### 3.1. Isolation and identification of bacterial strains

This study has been performed using a collection of 75 genetically unique enterococcal strains isolated from feces of mice fed four different diets (SD, BT, EVOO, and ROO) during 12 weeks. Strains were screened for enterococcal phenotypic characteristics and Gram-positive cocci, facultative anaerobic, catalase negative, able to hydrolyze esculin in 40% bile salts and to grow from 10 to 45°C, in a media containing 6.5% NaCl or buffered at pH 9.6, were selected and their r16S gene was sequenced. Fifty of these isolated have been reported and described elsewhere (21) and the rest are shown in Table 1, ascribed most of them to the species *E. faecalis* (12 strains) and *E. gallinarum* (9 strains), followed by *E. hirae* (4 strains). Nine strains were obtained at the beginning of the experiment, and eight both after six and twelve weeks. Eleven strains were obtained in the BT group, 10 in the EVOO group and four in the ROO group.

**Table 1:** Detection of virulence factors and biogenic amine gene PCR products in our *Enterococcus* collection.

		Virulence factors	Biogenic amines
Species		*esp*	*tdc*
0B1-1	*Enterococcus gallinarum*	+	
0B1-3	*Enterococcus faecalis*		
0B1-4	*Enterococcus hirae*		+
0B2-1	*Enterococcus gallinarum*	+	
0B2-3	*Enterococcus hirae*	+	
0B3-3	*Enterococcus faecalis*		+
0B3-4	*Enterococcus faecalis*	+	+
0O6-2	*Enterococcus hirae*	+	
0V3-5	*Enterococcus hirae*	+	
6B2-2	*Enterococcus hirae*		+
6B3-4	*Enterococcus faecalis*		+
6O5-1	*Enterococcus faecalis*		+
6O6-3	*Enterococcus faecalis*		+
6O7-2	*Enterococcus faecalis*	+	+
6V3-3	*Enterococcus faecalis*		+
6V5-3	*Enterococcus faecalis*		+
6V6-2	*Enterococcus gallinarum*	+	
12B1-1	*Enterococcus gallinarum*	+	
12B2-3	*Enterococcus gallinarum*	+	
12V3-1	*Enterococcus gallinarum*		
12V3-3	*Enterococcus faecalis*		+
12V5-1	*Enterococcus casseliflavus*	+	
12V5-3	*Enterococcus faecalis*		+
12V6-1	*Enterococcus gallinarum*	+	
12V6-2	*Enterococcus faecalis*	+	+

**Figure 1:**
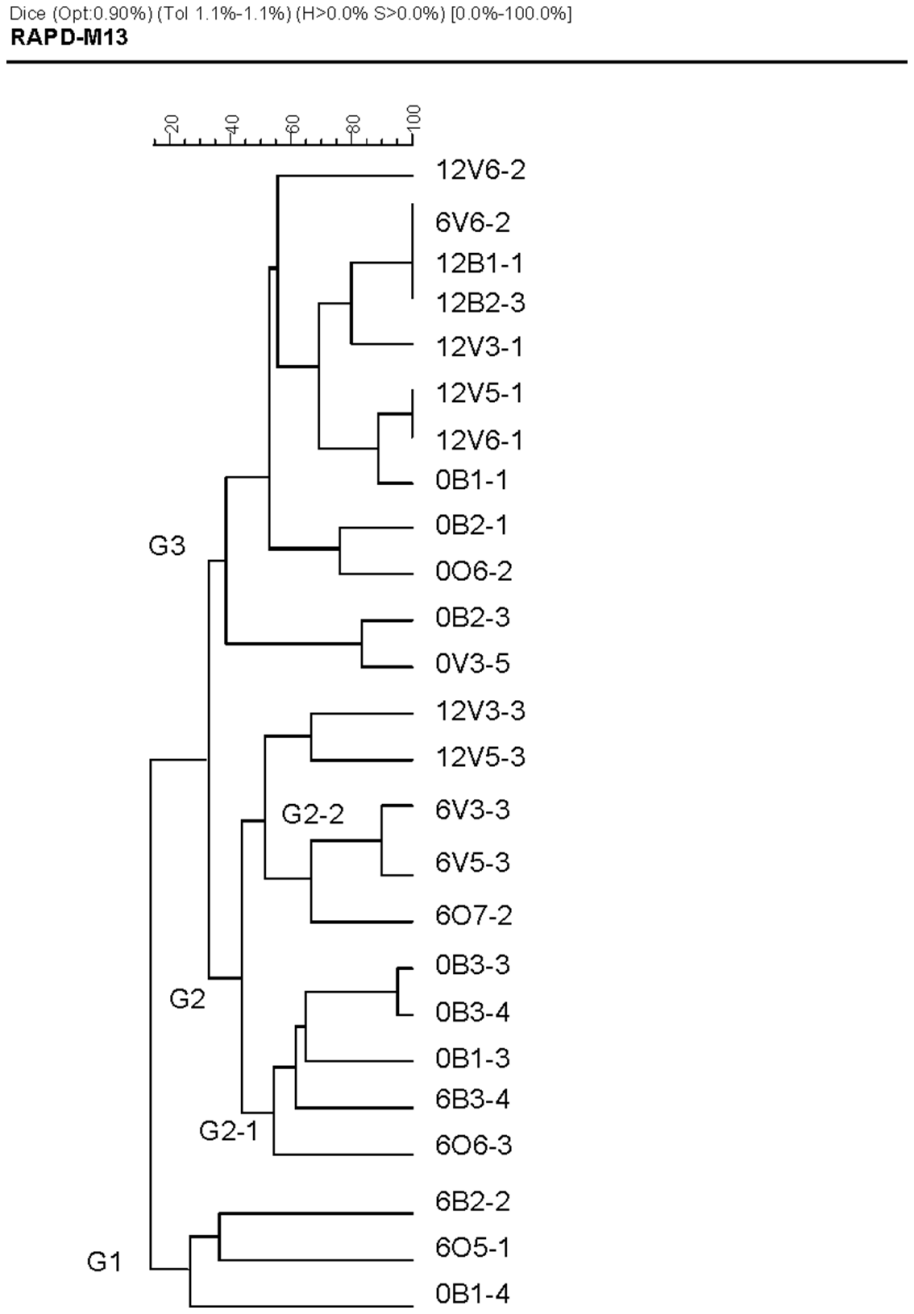
Pearson coefficient-based analysis of the RAPD profiles of the strains isolated.

In order to ensure the genetic uniqueness of each strain, all new isolates were subjected to a RAPD-PCR genotyping. All isolates from the same mouse and with the same profile were considered the same strain. Figure 1 shows the dendrogram with the genetic profile of the definite 25 newly characterized ones. Strains were clustered according to the species they belong and no pattern following timepoints or diets could be appreciated.

**Figure 2:**
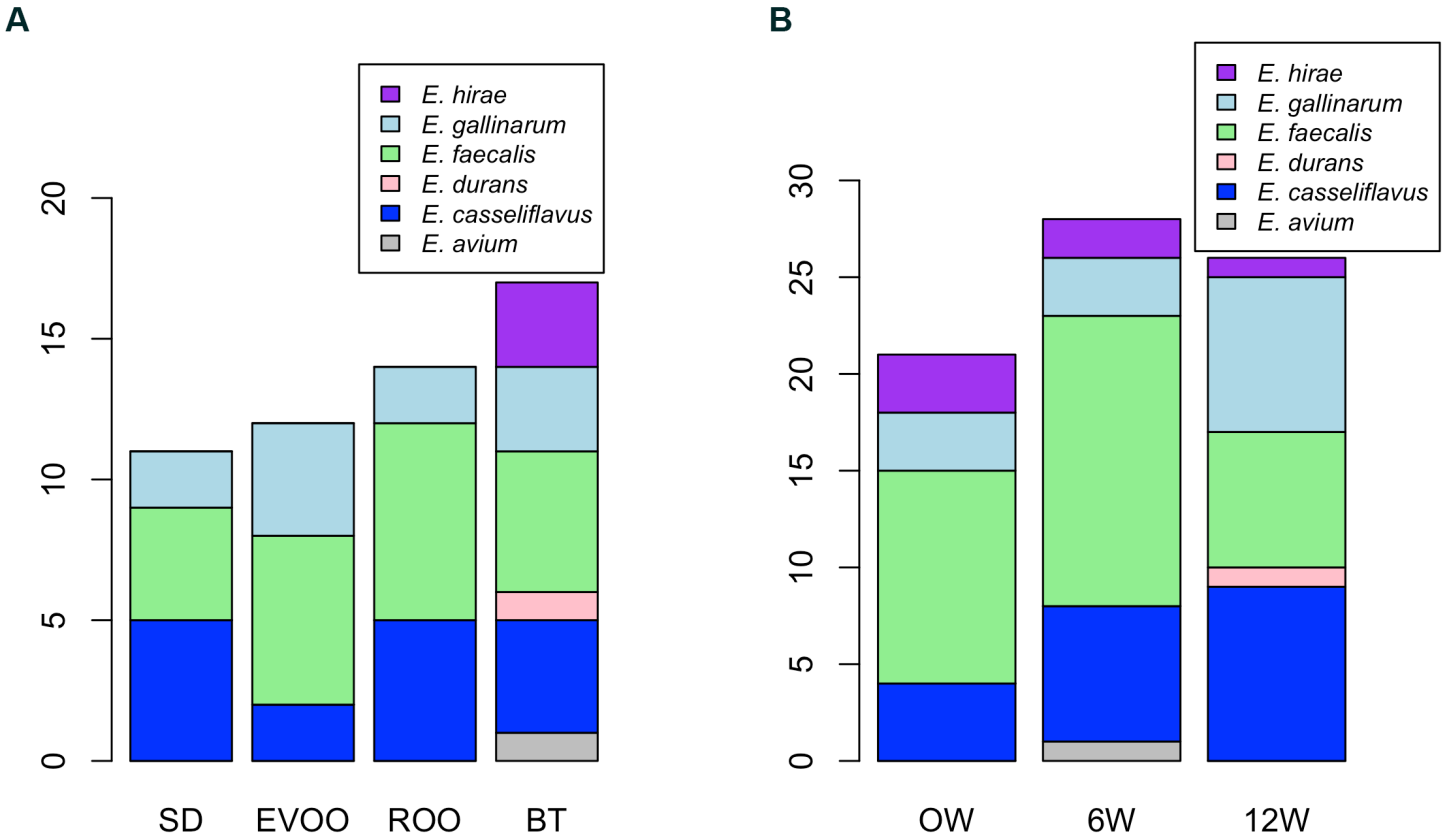
Bar plot of number of strains isolated within each species distributed according to diet **(A)** and time **(B)**. BT, Butter-enriched diet; ROO, diet enriched in refined olive oil; EVOO, diet enriched in extra virgin olive oil; SD, standard diet.

All 75 strains were considered together in order to uncover any specific different distribution, either across diets or time points. However, no significant difference was found in the species distribution of the strains with respect either to diet or time when applied a Fisher’s exact test for count data (Figure 2).

### 3.2. PCR amplification of virulence factors

**Figure 3:**
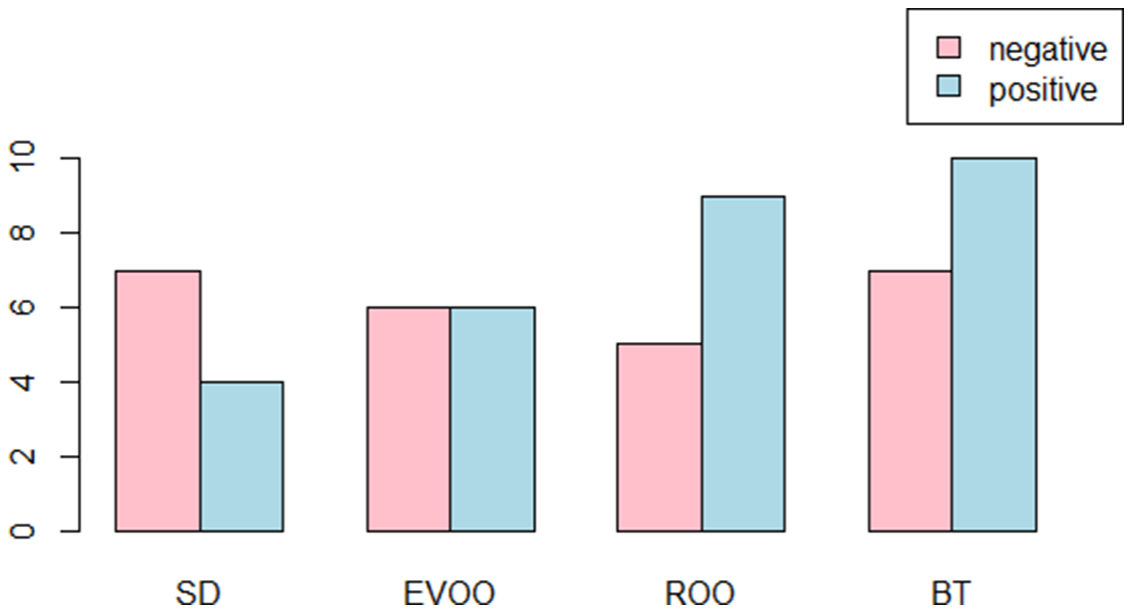
Bar plot of number of strains with presence (positives) or absence (negatives) of the gene *esp*, distributed among the four diets. BT, Butter-enriched diet; ROO, diet enriched in refined olive oil; EVOO, diet enriched in extra virgin olive oil; SD, standard diet.

Specific PCR reactions were carried out for detection of the presence of genes related to virulence factors. Eleven virulence factors were evaluated but none of the 25 strains were positive for them, except in the case of the gene coding for the enterococcal surface protein (*esp*) which was present in 52% of the strains (Table 1). In Figure 3 the number of positive and negative strains isolated under each diet are depicted. The number of positive strains is higher than the number of negative ones in BT and ROO, is equal in EVOO and lower in SD. However, no significant difference is obtained after contingence tables and a Fisher’s exact test are applied, although the *p* value is lower when SD is compared to ROO and BT together (*p* = 0.1795). No significant test or pattern was found with respect to time.

### 3.3. Biogenic amine production

In order to determine the possible production of biogenic amines, PCR amplification of the genes coding for the corresponding carboxylases were performed. No amplification product was obtained for the genes *hdc* and *odc*, but more than half the strains were positive for the gene coding for tyrosine descarboxylase (*tdc*) (Table 1).

**Figure 4:**
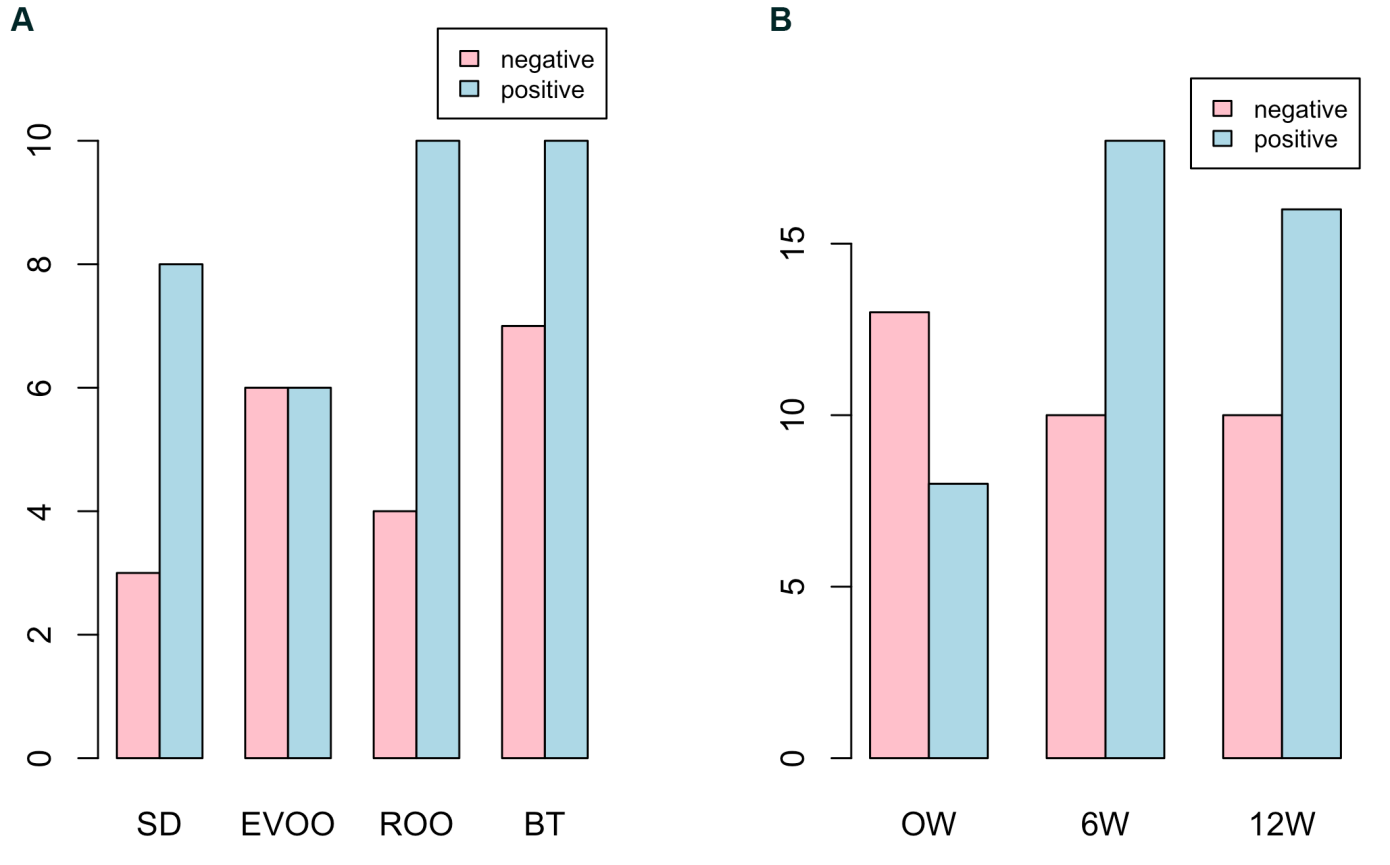
Bar plot of the number of strains with presence (positives) or absence (negatives) of the gene *tdc*, distributed among the four diets **(A)** or at the 0, 6 and 12 weeks from the beginning of the experiment considering strains from all diets **(B)**. BT, Butter-enriched diet; ROO, diet enriched in refined olive oil; EVOO, diet enriched in extra virgin olive oil; SD, standard diet.

When contingent tables followed by the corresponding Fisher’s test were performed, no significant differences were found in the distribution of positive and negative strains with respect to the diet (Figure 4A, *P* = 0.6422). In the case of the distribution with respect to the timepoints (Figure 4B), a change of tendency can be observed from 0 weeks (with a higher number of negative strains) to 6 and 12 weeks, where the trait is inverted. However, this difference is not enough to render a significant Fisher’s test (*p* = 0.1598). The tendency increases when the contingent table is pictured comparing 0 weeks with the other two timepoints together (*p* = 0.0706) and becomes significant (*p* = 0.0415) if strains from the EVOO group are not considered in the analysis.

### 3.4. Antibiotic resistance

Resistance antibiotic level was evaluated for 24 antibiotics and results were marked as R (resistance), I (intermediate) or S (susceptible) (Table 2).

**Table 2:** Antibiotic resistance.

Antibiotic resistance																								
	PENP	PENS	AMPE	CTXP	CTXS	IMIE	KAHES	GEHES	FQPR	MXFPS	LVXS	LVXP	ERYPS	TELPS	QDAE	TETPS	RFAPS	TSU	LNZEP	LNZS	FOSP	FURES	VAN	TEC
0B1-1	I	I	S	R	R	S	S	S	S	R	R	R	R	S	I	S	R	S	S	I	R	S	R	S
0B1-3	R	I	S	R	R	S	S	S	S	R	R	S	R	S	I	S	R	S	S	I	R	S	S	S
0B1-4	I	I	S	R	R	S	S	S	S	R	S	S	S	S	S	S	S	S	S	I	R	S	S	S
0B2-1	I	I	S	R	R	S	S	S	S	R	R	S	R	S	I	S	R	S	S	I	R	S	S	S
0B2-3	I	I	S	R	R	S	S	S	S	R	R	R	R	S	I	S	R	S	S	I	R	S	S	S
0B3-3	I	I	S	R	R	S	R	R	R	R	R	R	R	R	R	S	R	R	R	R	R	R	R	S
0B3-4	I	I	S	R	R	S	S	S	S	R	R	R	R	S	R	S	R	S	S	S	R	S	R	S
0O6-2	R	I	S	R	R	S	R	S	R	R	R	R	R	S	I	R	R	S	S	I	R	S	S	S
0V3-5	I	I	S	R	R	S	S	S	S	S	R	R	R	S	I	S	R	S	S	I	R	S	S	S
6B2-2	R	R	R	S	R	S	R	R	R	R	R	R	R	R	R	R	R	S	S	R	R	R	R	R
6B3-4	I	I	S	R	R	S	R	R	R	R	R	R	R	R	R	R	R	R	S	R	R	S	R	S
6O5-1	I	I	S	R	R	S	I	I	R	R	R	R	R	S	R	S	R	R	S	I	R	S	R	S
6O6-3	I	I	S	R	R	S	S	S	R	R	S	S	R	S	R	R	R	R	S	I	R	S	R	S
6O7-2	I	I	S	R	R	S	S	S	R	R	R	S	R	S	R	S	R	R	S	R	R	S	R	S
6V3-3	R	I	S	R	R	S	R	R	R	R	R	I	R	S	R	R	R	R	R	R	R	S	R	R
6V5-3	I	I	S	R	R	S	S	S	R	R	R	R	R	S	R	R	R	R	S	R	R	S	R	S
6V6-2	I	I	S	R	R	S	S	S	S	S	S	S	R	S	I	S	R	S	S	S	R	S	R	S
12B1-1	I	I	S	R	R	S	S	S	S	S	R	S	R	S	S	R	R	S	R	S	R	S	S	S
12B2-3	I	I	S	R	R	S	S	S	S	R	R	R	R	S	I	S	R	S	S	I	R	S	S	S
12V3-1	I	I	S	R	R	S	S	S	S	S	S	S	R	S	I	S	R	S	R	I	R	S	S	S
12V3-3	I	I	S	R	R	S	R	R	R	R	R	R	R	R	R	R	R	R	R	R	R	R	R	R
12V5-1	I	I	S	R	R	S	S	S	S	R	R	R	R	S	R	R	R	R	R	R	R	R	R	R
12V5-3	I	I	S	I	S	S	R	R	S	R	R	S	R	R	R	R	R	R	R	R	R	R	R	S
12V6-1	I	I	S	R	R	S	S	S	S	S	R	S	R	S	I	S	R	S	R	S	R	S	S	S
12V6-2	I	I	S	R	R	S	I	I	R	R	R	I	R	R	R	R	R	R	R	R	R	R	R	R

R, Resistant; I, Intermediate; S, Susceptible antibiotics. PENP, penicillin pneumo; PENS, penicillin strepto; AMPE, ampicillin; CTXP, cetofaxim pneumo; CTXS, cetoaxim strepto; IMIE, imipenem; KAHES, kanamycin; GEHES, gentamycin; FQPR, norfloxacin; MXFPS, moxifloxacin; LVXS, levofloxacin strepto; LVXP, levofloxacin pneumo; ERYPS, erythromycin; TELPS, telithromycin; QDAE, quinupristin-dalfopristin; TEPS, tetracyclin; RFAPS, rifampicin; TSU, cotrimoxazol; LNZEP, linezolid pneumo; LNZS, linezolid strepto; FOSP, fosfomycin; FURES, nitrofurantoin; VAN, vancomycin; TEC, teicoplanin.

**Figure 5:**
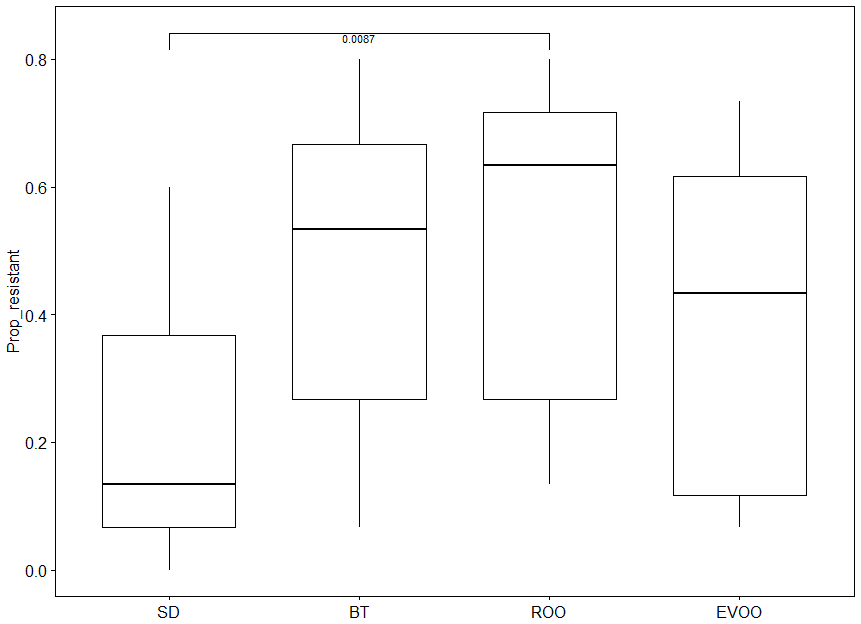
Boxplots of percentage of antibiotic resistances in each strain grouped by diet considering all time-points (*p* = 0.0114). BT, Butter-enriched diet; ROO, diet enriched in refined olive oil; EVOO, diet enriched in extra virgin olive oil; SD, standard diet.

The percentage of antibiotics to which each strain was resistant was calculated and these values were analyzed and compared by diets and by time-point using a Kruskal-Wallis test. No difference was found when analyzing datapoints but when the diets were compared significance differences were found (*p* = 0.0114) (Figure 5) and pairwise comparisons using Dunn’s test uncovered significant differences between SD and ROO (*p* = 0.0087) with a 90% signification also in the pair SD/BT (*p* = 0.0621).

### 3.5. Growth in the presence of polyphenols

**Figure 6:**
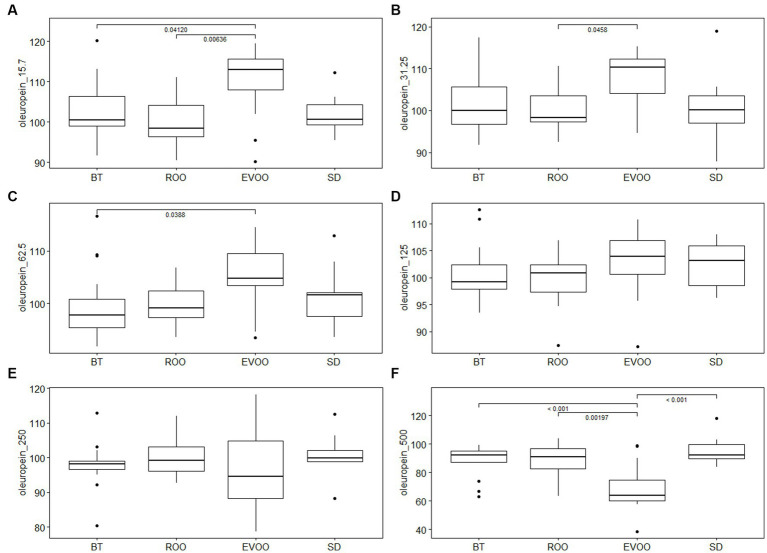
Box plot of growth percentage of strains isolated from mice fed different diets, under different concentrations of oleuropein: **(A)** 15.7 μg/mL; **(B)** 31.25 μg/mL; **(C)** 62.5 μg /mL; **(D)** 125 μg/mL; **(E)** 250 μg/mL and **(F)** 500 μg/mL. BT, Butter-enriched diet; ROO, diet enriched in refined olive oil; EVOO, diet enriched in extra virgin olive oil; SD, standard diet.

**Figure 7:**
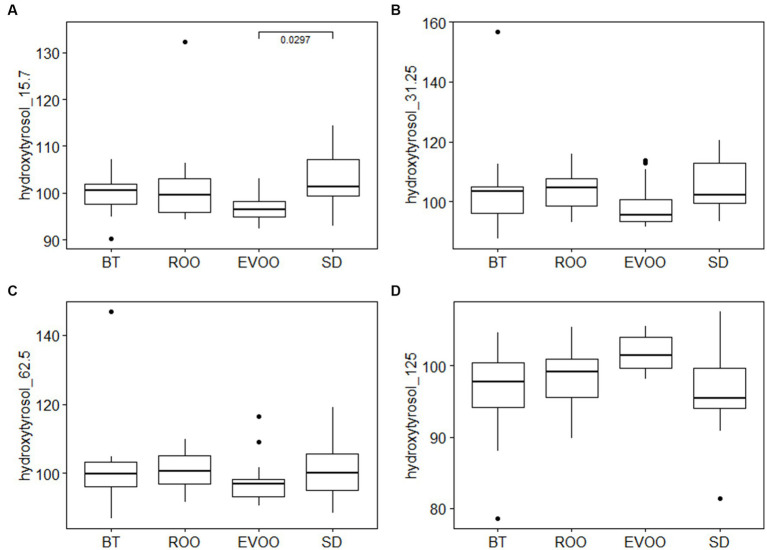
Box plot of growth percentage of strains isolated from mice fed different diets, under different concentrations of hydroxytyrosol: **(A)** 15.7 μg/mL; **(B)** 31.25 μg/mL; **(C)** 62.5 μg/mL and **(D)** 125 μg/mL. BT, Butter-enriched diet; ROO, diet enriched in refined olive oil; EVOO, diet enriched in extra virgin olive oil; SD, standard diet.

In order to find out if EVOO polyphenols affected the intestinal isolates differently depending on the host’s diet, the 75 strains were tested to grow in the presence of oleuropein (Sigma) or hydroxytyrosol (Sigma) at final concentrations of 15.7 μg/mL, 31.25 μg/mL, 62.5 μg /mL, 125 μg/mL, 250 μg/mL and 500 μg/mL. Strong inhibitions of the growth of the strains were not observed except at high concentrations of polyphenols (Supplementary Figures S1, S2). However, when carrying out a comparative statistical study using Kruskal-Wallis analysis in the 75 strains of the collection according to the different diets and at each of the concentrations, we have found significant differences between the strains isolated from mice fed a diet enriched with virgin olive oil compared to the other diets, both in the case of oleuropein (Figure 6) and with hydroxytyrosol (Figure 7). In the case of oleuropein, at low concentrations, strains isolated in the EVOO group fared better than the rest, as expected. However, at higher concentrations the statistical significance was lost and all strains were equally inhibited and, at the highest concentration, the tendency even inverted and EVOO strains grew significantly less than each of the other groups, as indicated by pairwise comparisons after performing Tukey contrasts.

In the case of hydroxytyrosol (Figure 7), the observed trend was opposite to the experiment with oleuropein, with lower growth in the EVOO group, although significant differences were only found at 15.7 μg/mL (value of *p* = 0.03891) while at 31.25 μg/mL and 62.5 μg /mL, with a similar profile, the data dispersion gave place to lack of significance (*p* = 0.415 and 0.5047 respectively). At 125 μg/mL, the other diets were more inhibited, which made EVOO show significant higher values, *p* = 0.039 (although the adjusted pairwise significance SD/EVOO was somewhat lower, *p* = 0.059). In higher concentration, the data was too dispersed and no differences at all could be detected among the diets.

## 4. Discussion

The strains we worked with in this study were obtained from the feces of mice fed a standard diet or one enriched with butter, extra virgin olive oil, or refined olive oil. The 75 isolates obtained were subjected to genetic profiling to ensure that the same strain was not isolated from the same mouse multiple times. The 75 isolates belonged to six different species of enterococci, and no significant species-specific distribution was found between diets or times, although three species were only present in the butter group (*E. hirae*, *E. avium*, and *E. durans*) and one of these three was only isolated at the end of the experiment (*E. durans*).

All strains were subjected to characterization with respect to the presence of virulence factors, production of biogenic amines and resistance to antibiotics. In previous works we had verified that the presence of the gene that encodes the enterococcal surface protein (*esp*) was more likely to be present in strains isolated from the group of mice fed high-fat diets (21). The result was supported by a significant Fisher’s test applied in contingency tables and it did not seem surprising since this protein promotes the primary binding to surfaces (23) and so, the strains that carried it could be supposed to have a selective advantage in an environment with a high influx of fat that could hinder the attachment to the mucosa. As can be seen from the current results expressed in Figure 3, there are certainly more negative strains than positive ones among the isolates from the standard diet group while the ratio is reversed in the case of diets high in butter or refined oil. In the EVOO group, the two types of strains have similar numbers, therefore EVOO shows a behavior closer to the standard diet than the other two diets, a circumstance that has been common in many studies (10, 24). It is noteworthy that, in the publication by Sánchez et al. (21), this diet lacked a significant number of strains, so it is now when we can have a better idea of how they behave. Perhaps partly because of this, the difference between diets is not significant when applying the Fisher test on this occasion, and consequently the trend in this regard does improve when we compare only the SD group with BT and ROO (*p* = 0.1795). In any case, the results of this specific study are compatible with the fact that the diet can promote the natural selection of a certain type of strains to colonize the host.

The presence of decarboxylases is another important undesired trait to check in enterococci, since they produce biogenic amines through the decarboxylation of amino acids (25) and the production of large amounts of these compounds can give rise to various toxicological risks (26). In our strains, the only decarboxylase gene found, expanding 52% of the isolates, was *tdc*, which codes for tyrosine decarboxylase (27, 28). Contrary to what happened in the case of the gene *esp.,* no differences were observed between the SD diet and the BT or ROO diets, with more positive than negative strains in all cases. However, once again the EVOO group stays out of this trend, with the same number of positives and negatives. Although no differences were found in the presence of *tdc* in the different diets, a strong tendency to increase with respect to time was detected, with a significance of 90% when comparing the initial time against the other two (*p* = 0.0706). Once again, the group of strains isolated from EVOO depart from this trend, as demonstrated by the fact that, if we remove this group from the analysis, Fisher’s exact test becomes 95% significant (*p* = 0.0415). These results improve the significance obtained previously (21) and it is important because, as we said at the time, it could turn out to be a trait related to the age of the host, with implications for their health since tyramine has been linked to hypertension and with an increase in blood glucose (29).

The third safety characterization that our strains underwent was on antibiotic resistance. Again, the strains were compared with each other with respect to the diet from which they came and the time at which they were isolated. For this, the percentage of resistance presented by each strain was made and the groups were compared using a Kruskal-Wallis test. In this case, no differences were found between the different times, but clear differences were found between diets, especially between the standard diet, with a lower percentage of resistance, and the refined olive oil and butter diets, in the latter case at 90%. For the third time, EVOO does not participate in this difference despite being a high-fat diet as well.

These results are important since they show a less harmful behavior of EVOO in relation to the other high-fat diets tested. The only feature that the BT and ROO diets, with different degrees of saturation from each other, have in common and that in turn differentiates them from EVOO, is the absence of an unsaponifiable fraction enriched in polyphenols, known for their selective antimicrobial action (11, 30). For this reason, the next step in this work was to study whether there were *in vitro* differences in the growth of the intestinal strains in the presence of EVOO polyphenols in order to check if the presence of these compounds was able to modulate the intestinal balance between strains of the same microbial group. It was reasonable to think that those strains isolated from an environment with a continuous supply of a product high in polyphenols would be more adapted to growing in their presence, which would demonstrate a prior selection in the intestine. However, the results obtained, even when they globally respond to this forecast, were more complex.

Certainly, in the case of oleuropein, while the other groups of isolates, both SD and high-fat diets, had similar growths to controls without polyphenol, isolates from mice fed EVOO had statistically significant higher growth in both at 15.7 (Figure 6A) as at 31.25 (Figure 6B) and 62.5 μg/mL (Figure 6C). However, this difference disappears and even eventually reverses at higher concentrations. At 125 μg/mL, the EVOO group still has a higher mean value, although it is no longer significant (Figure 6D). At 250 μg/mL (Figure 6E) the average value of the EVOO strains is already lower than the other diets, although still without statistical signification that however it is achieved at the highest concentration, 500 μg/mL (Figure 6F). At this point the strains of the EVOO diet present greater inhibition than the others, with statistically significant pairwise differences with each and every one of the remaining diets. This evolution of the effect of oleuropein on EVOO strains, from being a growth promoter at the lowest concentration, to becoming an inhibitor at the highest concentration, recalls the model of a saturation curve where microorganisms benefit from a substrate until they are overloaded and the undegraded excess, or a metabolite of it, accumulates and becomes pernicious. It is important to note that *in vitro* measurements reflect a very different reality from that of the intestinal ecosystem where bacteria grow naturally. In the intestine, the deleterious product may be diluted in the lumen, digested by other microbial species, or absorbed through the host’s mucosa, so it may not reach high concentrations. On the contrary, in the laboratory cultures this compound accumulates irremissibly increasing its concentration.

Oleuropein is a relatively larger compound that is metabolized to hydroxytyrosol and eleanolic acid (31). We do not have enough data to know what happens in cultures, but the fact that EVOO strains are inhibited in the presence of low concentrations of hydroxytyrosol is consistent with this compound being the harmful metabolite. The different size and polarity of oleuropein and hydroxytyrosol probably imply dissimilar mechanisms of absorption. Oleuropein, usually in the form of a glycoside, is likely to be absorbed *via* a glucose transporter (32), while hydroxytyrosol might get in *via* passive diffusion (33). This implies an active mechanism on the part of the bacterium to introduce the first compound into the cell, while the second would cross the membrane freely into the culture medium until concentrations on both sides were equal. At that time, it would begin to accumulate inside the cell as well, thus causing the observed damage. According to this analysis, strains from other diets would not metabolize oleuropein and therefore would not accumulate hydroxytyrosol in any case.

If this reasoning is true, it would be expected that, in the presence of hydroxytyrosol, the EVOO strains are inhibited (Figures 7A–C) but not that they do so to a greater extent than the strains of the other diets, or even less that eventually they hold up better at higher concentrations (Figure 7D). It is possible that the isolates of the EVOO group are adapted to stand or even metabolize minimal amounts of intracellular hydroxytyrosol since, as has been commented, it is very probable that it does not accumulate in the intestine. However, this may not occur in the hydroxytyrosol-unadapted strains from other diets, which need to have greater protection from the entry of the compound even at lower concentrations, protection that the EVOO strains would lack. But, in any case, both the oleuropein and hydroxytyrosol experiments are indicating that the isolates from EVOO-fed hosts behave clearly differently from the others, implying that there has been a prior selection in the intestinal ecosystem caused by the presence of the polyphenols of this type of oil.

If we add to this that these same EVOO strains have less resistance to antibiotics, a lower tendency to present virulence factors and that they do not participate in the dynamics of the other isolates to produce more biogenic amines over time, we can conclude that we are probably detecting a clear case of modulation of beneficial intestinal strains by diet. It is important to highlight that we are not talking about different genera, or sometimes not even different species (34) but about a much more subtle balance between genetically very close strains that, however, can make a difference in the health and the well-being of the host.

## Data availability statement

The original contributions presented in the study are included in the article/Supplementary material, further inquiries can be directed to the corresponding author.

## Ethics statement

The animal study was approved by University of Jaén Bioethics Committee. The study was conducted in accordance with the local legislation and institutional requirements.

## Author contributions

NA-T: Formal analysis, Investigation, Writing – original draft, Writing – review & editing. AC: Formal analysis, Investigation, Writing – review & editing. AM-R: Data curation, Software, Writing – original draft, Writing – review & editing. MH: Formal analysis, Investigation, Writing – review & editing. IP: Conceptualization, Funding acquisition, Resources, Writing – review & editing. AG: Resources, Writing – review & editing. MM-C: Conceptualization, Funding acquisition, Methodology, Project administration, Resources, Supervision, Validation, Visualization, Writing – original draft, Writing – review & editing.
