# Supplementary material for: Intestinal microbiota modulation at the strain level by the olive oil polyphenols in the diet
Source: Front Nutr. 2023 Oct 4;10:1272139. doi: 10.3389/fnut.2023.1272139 (PMC10582982; doi:10.3389/fnut.2023.1272139)
Supplement: Supplementary file 1 [file Data_Sheet_1.DOCX]

Supplementary Material

Intestinal microbiota modulation at the strain level by the olive oil polyphenols in the diet

**Natalia Andújar-Tenorio, Antonio Cobo, Ana Mª Martínez-Rodríguez, Marina Hidalgo, Isabel Prieto, Antonio Gálvez, Magdalena Martínez-Cañamero^*^**

*** Correspondence:** Magdalena Martínez Cañamero: canamero@ujaen.es

## Supplementary Figures

**
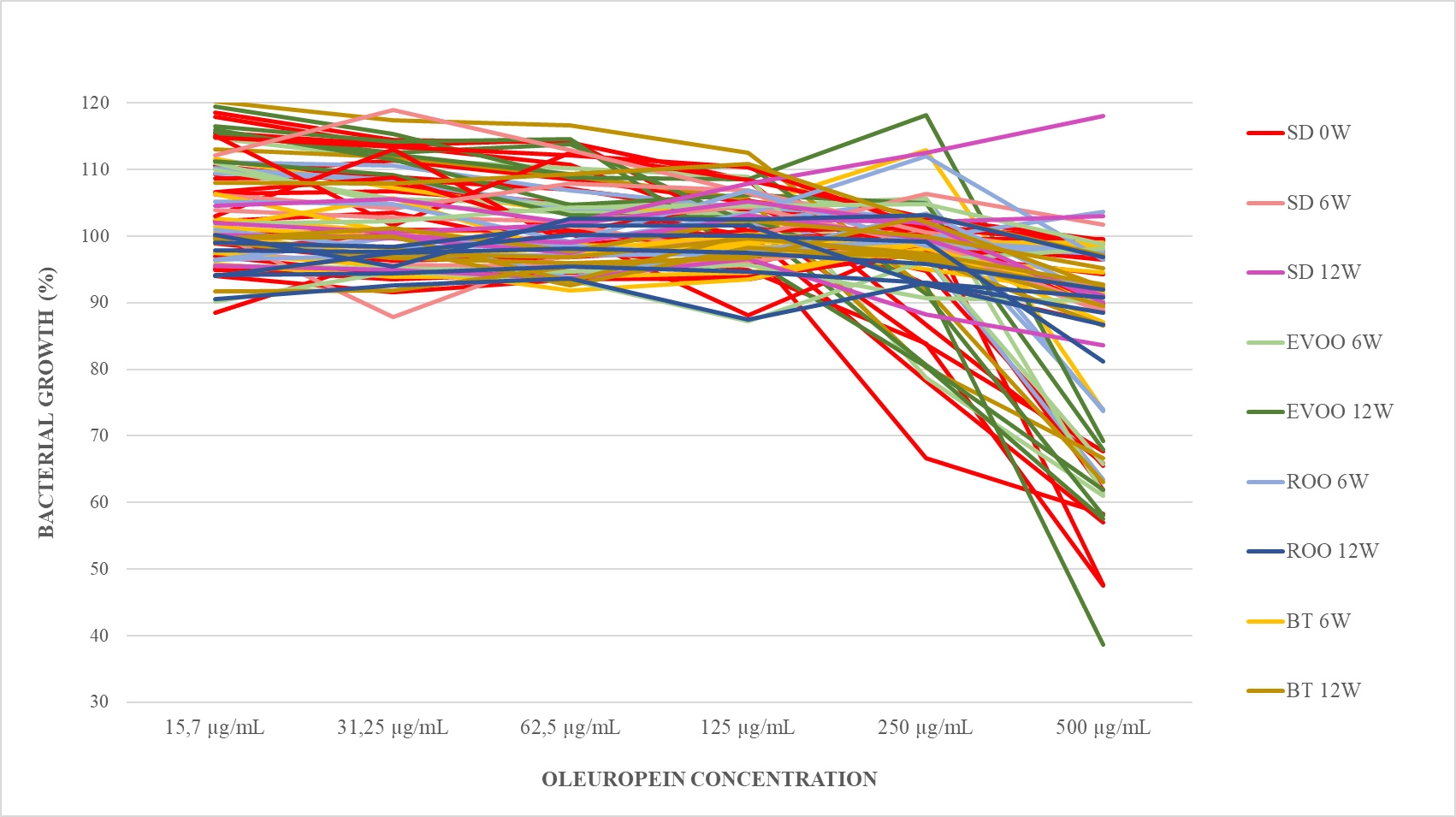
**

**Supplementary Figure 1.** Global results of the growth percentage of the enterococci strains in the presence of oleuropein with respect to a control without polyphenol. BT, Butter-enriched diet; ROO, diet enriched in refined olive oil; EVOO, diet enriched in extra virgin olive oil; SD, standard diet.

**
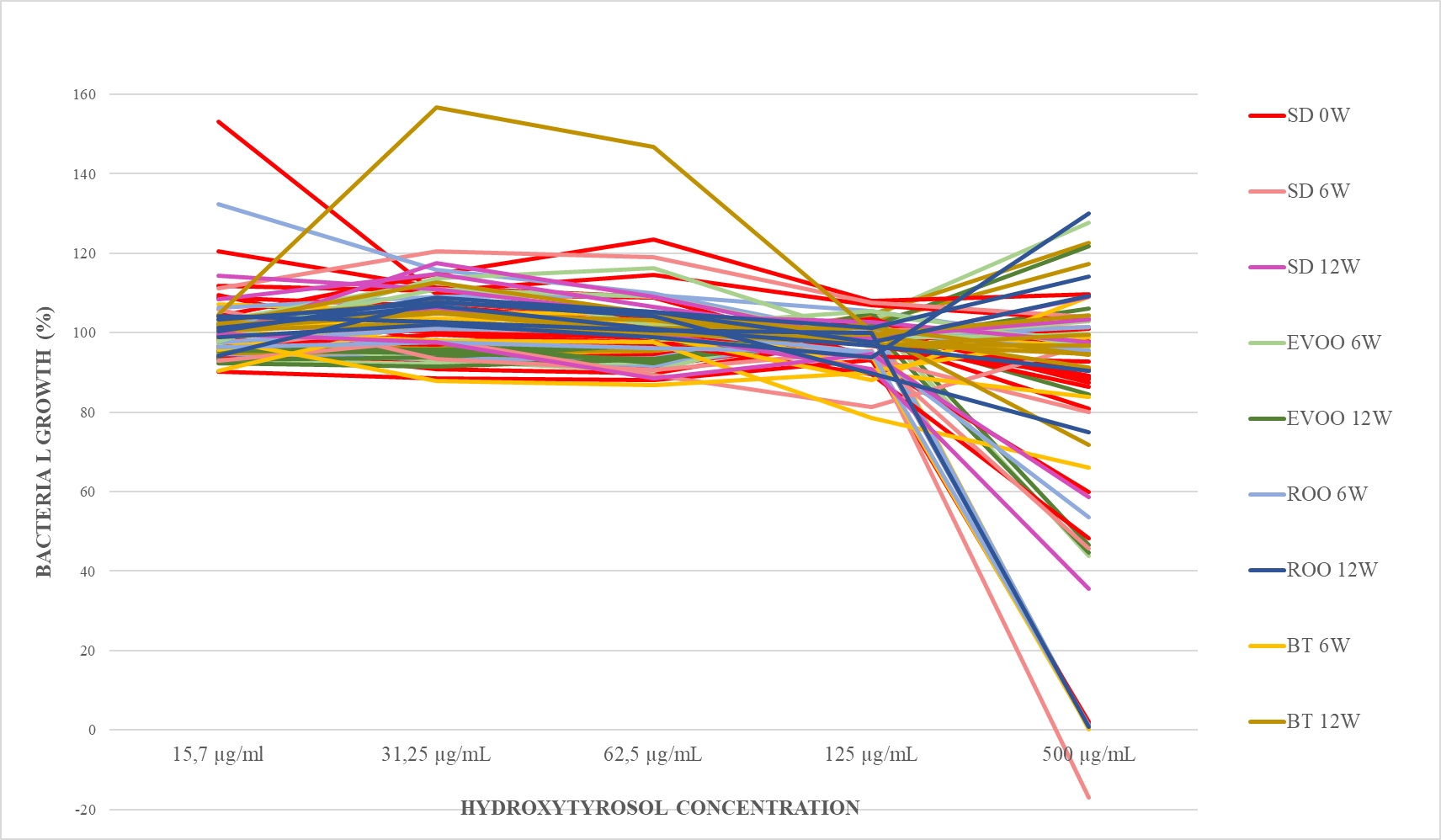
**

**Supplementary Figure 2.** Global results of the growth percentage of the enterococci strains in the presence of hydroxytyrosol with respect to a control without polyphenol. BT, Butter-enriched diet; ROO, diet enriched in refined olive oil; EVOO, diet enriched in extra virgin olive oil; SD, standard diet.
